# Supplementary material for: Pharmacotherapy problems and associated factors among type 2 adult diabetic patients on follow up at Mizan-Tepi University Teaching Hospital, Southwest Ethiopia
Source: PLoS One. 2023 Aug 4;18(8):e0288093. doi: 10.1371/journal.pone.0288093 (PMC10403119; doi:10.1371/journal.pone.0288093)
Supplement: S1 File — (DOCX) [file pone.0288093.s001.docx]

**Mizan-Tepi University, College of Medicine and Health Sciences**

**School of Pharmacy**

**Data Collection Tool/** **Questionnaire**

Dear Sir/madam

This data collecting format is prepared to collect data on “**Pharmacotherapy Problems and Associated Factors among Type 2 Adult Diabetic Patients on Follow up at Mizan-Tepi University Teaching Hospital, Southwest Ethiopia”**. The aim of the study is to assess drug therapy problems and associated factors among diabetic type 2 patients on follow-up at Mizan-Tepi University Teaching Hospital, Southwest Ethiopia.

We assure you that your answers are kept completely secured and confidential. The research is purely for academic purpose. Your genuine response to this question will help us for the correct finding of the study. The study result will not include name of participants and address.

We are really grateful in advance for your cooperation to be part of this study!!!

....….

Are you willing to continue? Yes: No:

**Part I: Socio-demographic characteristics of patients with type 2 diabetes mellitus on follow up at MTUTH, Southwest Ethiopia, 2022.**

1. Sex: A. Male B. Female
2. Age (year):--------------
3. Family history of DM A. yes B. no
4. weight----------- kg, height--------------meter BMI ---------kg/m2
5. marital status: A. single B. Married C. divorced D. Widowed
6. Educational status: A. informal education B. Primary education (1-8) C. secondary education (9-12) D. Higher Education
7. Occupational status: A. government employee B. Unemployed C. farmer D. merchant E. house wife G. other (specify)
8. Religion A. Orthodox B. Muslim C. protestant D. Other (specify) ____________
9. Ethnicity A. Oromo B. Amhara C. Tigre d. Bench E. other (specify) ____________
10. Social drug use (a). Alcohol (b) Chat (c).Tobacco (d). no

**Part II: Drug therapy problems (DTPs) among adult type 2 diabetes patients on follow up at MTUTH, Southwest Ethiopia, 2022.**

| **No.** | **Type of DTP** |  |
| --- | --- | --- |
| **1** | Need for additional drug therapy |  |
| **2** | Unnecessary drug therapy |  |
| **3** | Ineffective drug therapy |  |
| **4** | Dosage too low |  |
| **5** | ADR |  |
| **6** | Dosage too high |  |
| **7** | Noncompliance |  |

**Part III: Clinical and drug therapy conditions of adult type 2 diabetic patients on follow up at MTUTH, Southwest Ethiopia, 2022.**

| No. | Variable | | Characteristics |
| --- | --- | --- | --- |
| 1 | Organ function tests | Kidney | 1. Yes B. No |
|  |  |  | If yes, tests done and obtained results___________ |
|  |  | Liver | A, Yes B. No |
|  |  |  | If yes, tests done and obtained results_______________ |
| 2 | Presence of diabetes complications | | A. nephropathy B. retinopathy C. neuropathy D) Others______ |
| 3 | Numbers of complication per patient | |  |
| 4 | DM duration in year | | ≤ 4 |
|  |  |  | 5-9 |
|  |  |  | ≥ 10 |
| 5 | Presence of co-morbidity | | A. Yes B. No |
| 6 | yes, number of co-morbidity | |  |
| 7 | Type of co-morbidity | | Hypertension |
|  |  |  | Asthma |
|  |  |  | Dyspepsia |
|  |  |  | Dyslipidemia |
|  |  |  | Heart failure |
|  |  |  | Others |
| 8 | Number hospitalizations in last 1 year | |  |
| 9 | Duration on treatment | |  |
| 10 | Number of emergency visit in last 1 year | |  |
| 11 | Duration since diagnosis of type two DM | |  |
| 12 | Frequency of medication taken per day | |  |
| 13 | Total number of medications taken per day | |  |
